# Blinding peer review

**DOI:** 10.7554/eLife.74744

**Published:** 2021-11-24

**Authors:** Michael A Taffe

**Affiliations:** 1 Department of Psychiatry and the Skaggs School of Pharmacy, University of California, San Diego San Diego United States

**Keywords:** peer review, racial disparities, racial bias, funding, halo effects, None

## Abstract

Concealing the identity of the principal investigator only partially closes the success gap between white and African American or Black researchers in NIH grant applications.

**Related research article** Nakamura R, Mann LS, Lindner MDP, Braithwaite J, Chen MC, Vancea A, Byrnes N, Durrant V, Reed B. 2021. An experimental test of the effects of redacting grant applicant identifiers on peer review outcomes. *eLife* **10**:e71368. doi: 10.7554/eLife.71368

Every year, approximately 55,000 research grant applications are submitted to the US National Institutes of Health (NIH); of those only 11,000 will be selected for funding. NIH-funded research drives major advances in scientific knowledge, medicine and healthcare, helping to improve health, to reduce morbidity and to create economic innovation. Yet, how this funding is distributed is increasingly coming under scrutiny.

In particular, research shows that applications spearheaded by principal investigators who identify as African American or Black (AAB) do not get funded as often as those led by white researchers. From 2000 to 2006, for instance, Research Project Grant (R01) applications by AAB investigators were 42% less successful than those led by white researchers (Ginther et al., 2011). When the report highlighting this gap was first published in 2011, the Director of the NIH asserted that “the situation [was] not acceptable” (Corbyn, 2011); yet a similar 40 % reduction in success rate was reported for applications with AAB principal investigators submitted from 2011 to 2015 (Hoppe et al., 2019). AAB leaders were more likely to propose investigating questions that were less often awarded money, but they were also less likely to be funded regardless of grant topic.

Overall, this body of work suggests that AAB scientists have unequitable access to public resources, hindering the advance of knowledge – especially on health care topics of interest for communities of color (Dzirasa, 2020; Gilpin and Taffe, 2021; Harnett, 2020; Stevens et al., 2021; Taffe and Gilpin, 2021). These disparities shed light on biases that may contaminate the grant-awarding mechanism, spurring interest into whether the process could be improved. Now, in eLife, Bruce Reed and colleagues at the NIH/Center for Scientific Review – including Richard Nakamura as first author – report the impact of reviewer blinding on the funding gap (Nakamura et al., 2021).

Typically, each NIH application is first evaluated in depth by three peer scientists, who provide an initial ‘overall-impact’ score which is averaged to rank the submissions. A panel of 20–30 researchers then assembles to discuss the top half of the applications assigned to them; together, they vote on a final overall-impact score for each of these projects, following a discussion led by the three assigned reviewers. Finally, these scores are used by the 24 NIH Institutes and Centers that issue the grants to decide which projects to fund (Kienholz and Berg, 2013). Initial peer review therefore plays a major role in determining which applications will receive a grant by providing an all-critical preliminary impact score. Many have therefore proposed that blinding these reviewers to the identity of the applicants could help to potentially eliminate the disparity between white and AAB investigators.

To explore this question, Nakamura et al. obtained 400 R01 applications with AAB principal investigators submitted and reviewed in 2014–2015, as well as two comparison sets of 400 applications with white investigators. One of these two sets was randomly selected from the 26,000 applications submitted in the same period; the other was created by ensuring that the applications matched those with AAB principal investigators on several characteristics, including the preliminary impact score from the assigned reviewers. The team removed any identity information from the proposals. Both anonymized and original, unredacted applications were then peer-reviewed by different sets of researchers.

Results showed that anonymizing the applications reduced the scores for projects led by white investigators, but this manipulation did not change the scores of applications from AAB researchers. Overall, the reduction in white investigators’ scores only closed the AAB-white gap by about half. Yet, several methodological issues may limit how well these results could translate to actual NIH review processes.

First, reviewers did not meet in panels to discuss applications and vote final scores; a critical part of the NIH reviewing process was therefore not duplicated, and the impact of blinding this step cannot be determined. Second, Nakamura et al. report that 22% of reviewers ‘broke the blind’ by correctly identifying the specific principal investigator or research group leading the anonymized application. Merely removing direct identifiers from proposals may therefore not be sufficient to blind review. Finally, scores were not always replicated between the original and study reviews – they were improved for white investigators in the group matching AAB applicants’ scores. This discrepancy could imply that the reviews conducted for the study might have been done differently than during the original process.

Importantly, the work by Nakamura et al. shows that changing NIH grant evaluation to blinded review will have limited impact, one that will take place primarily through reducing the advantage of non-anonymized proposals for white investigators. White researchers from one of the groups received different scores in the study, compared to the original review, which clearly suggests that review outcomes at the NIH may not reflect objective and highly repeatable assessments of merit.

This study has critical implications for fixing the NIH funding disparity first identified in the 2011 Ginther report. So many have proposed that blinding review is a simple solution; Nakamura et al. have shown that this would be insufficient. This work, combined with identified disparities in funding of topics (Hoppe et al., 2019), should re-orient the NIH away from trying to identify singular causes, and towards applying direct fixes with immediate impact.
